# Analytical performance of OncoPrism-HNSCC, an RNA-based assay to inform immune checkpoint inhibitor treatment decisions for recurrent/metastatic head and neck squamous cell carcinoma

**DOI:** 10.1186/s12885-024-13362-8

**Published:** 2025-01-07

**Authors:** Jeffrey Hiken, Jon Earls, Kevin C. Flanagan, Rachel L. Wellinghoff, Michelle Ponder, David N. Messina, Jarret I. Glasscock, Eric J. Duncavage

**Affiliations:** 1 Cofactor Genomics, Inc, 4044 Clayton Ave, St. Louis, MO 63110 USA; 2 Department of Pathology and Immunology, Washington University School of Medicine, St. Louis, MO USA

**Keywords:** OncoPrism, Head and neck cancer, HNSCC, Analytical validation, Biomarker, Classifier, Assay

## Abstract

**Background:**

While immune checkpoint inhibitor (ICI) therapies can significantly improve outcomes for patients with recurrent/metastatic head and neck squamous cell carcinoma (RM-HNSCC), only about 15–20% benefit from such treatments. Clinical tests that guide the use of ICIs are therefore critically needed. OncoPrism-HNSCC was developed to address this need. The assay combines next generation RNA sequencing-based immunomodulatory gene expression signatures with machine learning algorithms to generate an OncoPrism score that classifies patients as having low, medium, or high likelihood of disease control in response to ICI treatment. Also, OncoPrism-HNSCC leverages the same FFPE patient tumor RNA used for ICI response prediction to identify rare cases where oncogenic rearrangements in *NTRK1/2/3* or *ALK* genes may occur, and which may indicate the use of potentially highly effective targeted therapies. The clinical performance of OncoPrism-HNSCC has been validated. Here, we report its analytical performance in the presence of potentially confounding sources of variation.

**Methods:**

The assay’s analytical sensitivity was assessed by varying RNA input quantity and quality, observing the effect on ICI response prediction scores. Analytical specificity was tested by spiking increasing percentages of genomic DNA into input RNA. Intra-assay and inter-assay precision were evaluated, and the analytical sensitivity, specificity, and precision of gene fusion detection were assessed. Concordance with orthogonal methods of gene fusion detection was tested on 67 FFPE clinical samples.

**Results:**

Varying RNA inputs as low as four-fold below the nominal input amount had little effect on ICI response prediction scores. RNA quality levels below the test threshold had no significant effect. Genomic DNA spike-ins up to 30% had only a small effect on scores. The pooled standard deviation for multiple operators, reagent lots, batches, and sequencers yielded an overall variance represented by just 0.87% of the score range of the test (0–100). *NTRK* and *ALK* gene fusion detection was 100% concordant with orthogonal methods.

**Conclusions:**

Robust and reliable analytical performance of the OncoPrism-HNSCC assay supports its clinical use, even in the presence of variation typically encountered in the laboratory setting.

**Supplementary Information:**

The online version contains supplementary material available at 10.1186/s12885-024-13362-8.

## Background

Head and neck squamous cell carcinoma (HNSCC) represents a significant healthcare burden worldwide. With nearly 900,000 new cases and 450,000 deaths annually, HNSCC is the sixth most common cancer globally, and its prevalence is expected to rise to 1.08 million cases annually by 2030 [1, 2]. The majority of HNSCC patients present with locoregional disease, but 50% eventually progress to recurrent or metastatic (RM) disease after curative-intent treatment [2]. Unfortunately, patients with RM-HNSCC face a poor prognosis, with median overall survival of around 1 year [3].

Immune checkpoint inhibitors (ICIs), such as pembrolizumab and nivolumab, which target programmed cell death protein 1 (PD-1), have become part of mainstream systemic treatments for RM-HNSCC [4]. While the advent of ICIs has provided significant improvements in the treatment of RM-HNSCC, the benefit accrues to only about 15–20% of patients [2]. The biomarker most widely used to inform treatment decisions about ICIs in RM-HNSCC is programmed death-ligand 1 (PD-L1) expression. The PD-L1 immunohistochemistry (IHC) test, however, does a poor job of predicting clinical benefit, as measured by Disease Control Rate (DCR, defined as the fraction of patients without disease progression, post-treatment). In KEYNOTE-048, patients with a PD-L1 IHC CPS ≥ 1 saw only a 3% enrichment in disease control in response to ICI monotherapy, and no enrichment in response to combination ICI-chemotherapy [5, 6]. While ICIs provide clear clinical benefit to a subset of patients, current methods for identifying which patients benefit are insufficient.

We previously described a laboratory developed test (LDT), OncoPrism-HNSCC, that predicts disease control with increased sensitivity and specificity compared to PD-L1 IHC for RM-HNSCC patients treated with ICIs [7, 8]. OncoPrism-HNSCC is an RNA-sequencing-based test that uses dimensionality reduction and machine learning to classify patients into three groups: low (low likelihood of disease control in response to ICI), medium (indeterminate likelihood of disease control in response to ICI), and high (high likelihood of disease control in response to ICI). The test predicts disease control in response to anti-PD-1 treatments with high sensitivity and specificity [7].

In addition to predicting ICI response, OncoPrism-HNSCC also identifies targetable gene rearrangements. Promising targeted therapies are emerging for patients whose tumors harbor certain somatic gene fusions and have shown effectiveness across numerous solid tumor types. For example, fusions involving the neurotrophic tyrosine receptor kinase genes NTRK1, NTRK2, and NTRK3 are biomarkers for the therapeutic use of selective inhibitors, such as larotrectinib and entrectinib, which show remarkably effective response rates, reportedly > 75% [9, 10]. Patients whose tumors possess rearrangements in anaplastic lymphoma kinase (ALK) can benefit, with high response rates, from the targeted tyrosine kinase inhibitors crizotinib and alectinib [11].

The prevalence of *NTRK* gene fusions is less than 1% across all cancers, and outside of non-small cell lung cancer, *ALK* gene fusions occur in about ∼ 0.2% of cancers [11, 12]. Most patient tumors are not tested for these fusion events, except in indications where they are most prevalent. OncoPrism-HNSCC reports fusions involving *NTRK* and *ALK* genes as Tier I variants, consistent with current AMP/ASCO/CAP guidelines [13]. Thus, OncoPrism-HNSCC provides guidance for common ICI treatment decisions and identifies the relatively rare patients who may benefit from less common, but potentially life changing, gene-fusion targeted therapies.

**Fig. 1:**
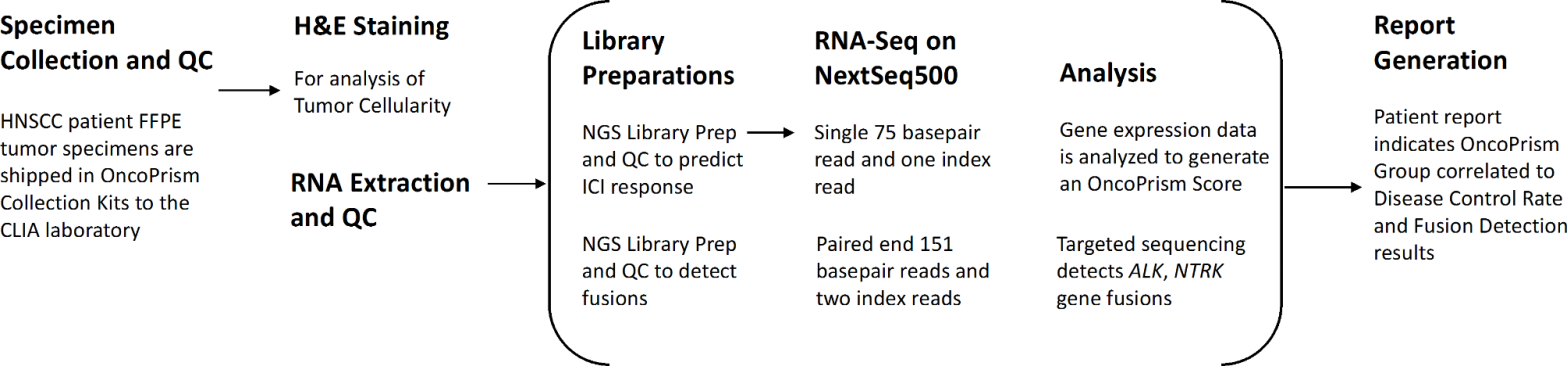
Workflow for the OncoPrism-HNSCC test

**Fig. 2:**
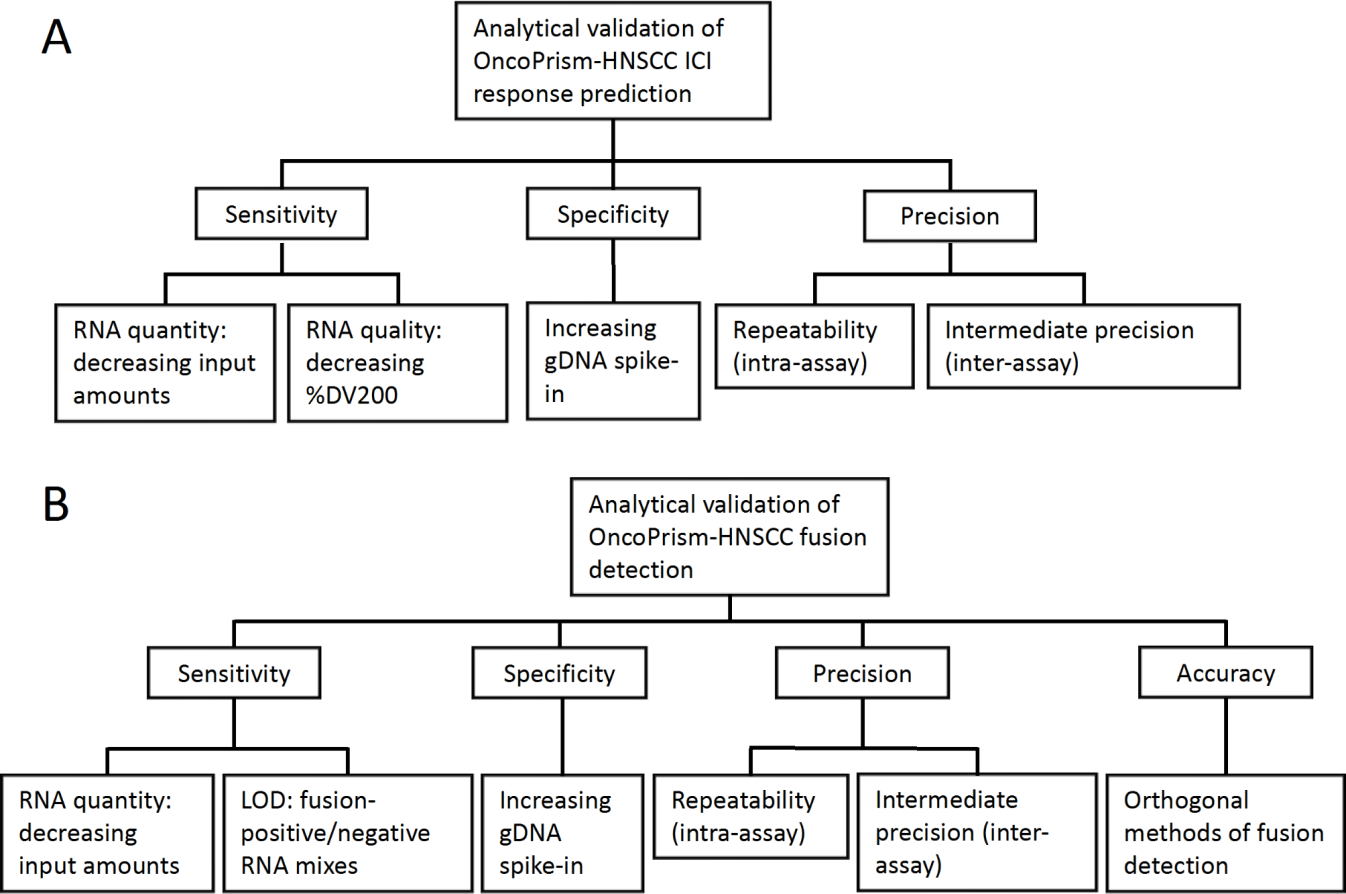
Experimental design for OncoPrism-HNSCC analytical validation. (**A**) ICI response prediction. (**B**) *ALK* and *NTRK* gene fusion detection

Before an LDT can be implemented in the clinical setting, the test must demonstrate its ability to produce reliable and consistent results under conditions that reflect day-to-day sources of variability in the laboratory setting. As part of the regulatory approval process, we followed the ACCE model for the technical assessment of prospective LDTs [14]. ICI response prediction by the OncoPrism-HNSCC assay (Fig. 1) has been clinically validated [7]. Here we evaluate the analytical validity of OncoPrism-HNSCC by testing its analytical sensitivity, specificity, repeatability, and intermediate precision (Fig. 2). For the ICI prediction component of OncoPrism-HNSCC, sensitivity was assessed by testing the effect of RNA input quantity and quality on OncoPrism scores and categorical calls. For specificity, we tested the effect of gDNA spike-ins into sample RNAs. For repeatability, we analyzed variation of OncoPrism-HNSCC test results when replicate samples were processed by a single operator using the same reagent lots and instruments. Intermediate precision was assessed by analyzing variation of OncoPrism-HNSCC test results when samples were processed with multiple operators, reagent lots, days, and instruments. Similarly, analytical validation of the *NTRK1/2/3* and *ALK* fusion detection component of OncoPrism-HNSCC included investigation of sensitivity, specificity, repeatability, and intermediate precision. In addition, the concordance of OncoPrism-HNSCC fusion detection compared with orthogonal methods of fusion detection was assessed using 67 clinical samples.

## Methods

### Validation study specimens

FFPE HNSCC clinical tumor samples were sourced from patients enrolled in the PREDicting immunotherapy efficacy from Analysis of Pre-treatment Tumor biopsies (PREDAPT) study (NCT04510129) [8]. All samples were biopsied prior to any anti-PD1 immune checkpoint inhibitor therapy. Additional FFPE well characterized head and neck cancer tumor samples were sourced from other laboratories. Additional FFPE tumor samples with known *ALK* and *NTRK* rearrangements were acquired from iSpecimen (Lexington, MA), Precision for Medicine (Frederick, MD), and Cureline (Cureline, Brisbane, CA). Available patient sample data is listed in Additional-file-1_patient-sample-data. A key indicating the analyses for which each sample was used is listed in Additional-file-2_sample-key.

### RNA extraction

RNA was extracted using the RNAstorm™ FFPE RNA Extraction Kit (Biotium, Fremont, CA) according to the manufacturer’s instructions. RNA quantity was assessed with the High Sensitivity RNA Qubit assay (Thermo Fisher Scientific, Waltham, MA). RNA quality was assessed using the Agilent Bioanalyzer 2100 (Agilent Technologies, Santa Clara, CA).

### NGS library preparation for ICI response prediction

RNAseq libraries for OncoPrism-HNSCC ICI response prediction were prepared using the QuantSeq 3’ mRNA-Seq Library Prep Kit FWD for Illumina (Lexogen, Inc., Greenland, NH), following the manufacturer’s instructions. Library RNA input was 40 ng total RNA for all samples unless otherwise specified. UMI Second Strand Synthesis Module for QuantSeq FWD (Lexogen, Inc., Greenland, NH) replaced Second Strand Synthesis Mix 1 in the workflow. These methods use an oligo-dT primer for First Strand Synthesis, resulting in libraries highly enriched for poly-adenylated transcripts. All samples were processed with two OncoPrism-HNSCC positive controls and a No Template Control. The positive (high or medium scoring) controls were RNA extracted from RM-HNSCC samples as described above. Final libraries were sequenced to a minimum depth of 5 million single-end 75 bp reads on a NextSeq500 (Illumina, San Diego, CA), following the manufacturer’s protocols.

### Processing of RNA sequencing data

FASTQ files were preprocessed with trim_galore/cutadapt version 0.4.1 to remove adapter sequences as well as reads with PHRED quality scores less than 20 and reads that were less than 20 basepairs. The trimmed reads were aligned to the human genome GRCh38 with STAR version 2.5.2a using the two-pass method as previously described [15]. Read counts were generated using htseq-count version 0.9.1 and annotation from Gencode version 22. The data was normalized as counts per million (CPM) and log2 transformed using unique reads aligning to protein coding regions. Samples were required to have a minimum of 30% exonic alignment and 800,000 unique deduplicated counts to be included in the study.

### OncoPrism-HNSCC ICI response prediction scores

The OncoPrism-HNSCC biomarker generates an OncoPrism score from 0 to 100 that correlates with predicted disease control in patients with RM-HNSCC treated with anti-PD-1 monotherapy [7]. Higher OncoPrism scores represent higher confidence by the model that the patient will have disease control. The thresholds for the OncoPrism groups were defined from training data. The threshold between the low group (OncoPrism scores 0–37) and the medium group (OncoPrism scores 38–51) is defined as the value of the 25th percentile mean score. The threshold between the medium group and the high group (OncoPrism scores 52–100) is defined as the value of the 50th percentile mean score. These training cohort mean score thresholds are used for all subsequent validation and analysis to define the OncoPrism groups.

### RNA fragmentation for %DV200 validation study

FFPE RNA with decreasing %DV200 values was generated using the NEBNext^®^ Magnesium RNA Fragmentation Module (New England Biolabs, Ipswich, MA). For each fragmentation time point, 500 ng RNA in a volume of 18 µl water was combined with 2 µl 10X RNA Fragmentation Buffer in thin-walled PCR tubes. Tubes were transferred to a preheated (94˚C) thermocycler and incubated for 0, 1, 2, 3, 4, or 5 min. Fragmentation was terminated by transferring tubes to an ice-cold aluminum block and immediately adding and mixing 2 µl 10X RNA Fragmentation Stop Solution. Fragmented RNA was purified using the Zymo RNA Clean & Concentrator-5 kit (Zymo Research, Irvine, CA) according to manufacturer’s instructions. %DV200 was calculated using an Agilent Bioanalyzer 2100 (Agilent Technologies, Santa Clara, CA).

### gDNA spike-in validation study

Genomic DNA from two different FFPE-preserved HNSCC tumor samples was extracted and pooled. The gDNA was extracted using the DNAstorm™ FFPE DNA Extraction Kit (Biotium, Fremont, CA) according to the manufacturer’s instructions. The gDNA extraction kit was sourced from the same vendor as the RNA extraction kit, and uses similar methodologies. DNA quantity was assessed with the High Sensitivity dsDNA Qubit assay (Thermo Fisher Scientific, Waltham, MA). The RNA input was held constant for RNAseq library preparations, and gDNA was spiked-in at increasing percentages on a per mass basis (0, 5, 10, 20, 30%).

### Gene fusion detection

Targeted anchored multiplex PCR libraries for fusion detection were prepared using the Archer™ FUSIONPlex™ Core Solid Tumor Panel kit and Archer MBC adapters (IDT, Coralville, IA), according to the manufacturer’s protocol. Libraries were quantified using the KAPA Library Quantification Complete kit (Universal; Roche, Indianapolis, IN) on a QuantStudio 3 qPCR machine (ThermoFisher, Waltham, MA). Libraries were sequenced to a minimum depth of 7 million paired-end 151 bp reads (3.5 million clusters) on a NextSeq500 (Illumina, San Diego, CA), following the manufacturer’s protocols. Gene fusions were identified using the Archer™ Analysis (v7.2) pipeline. Sequencing data was initially randomly subsampled to a depth of 3.5 million reads. To be reported as fusions, potential fusion events required at least 3 supporting start sites, at least 5 supporting reads, and at least 10% of unique fragments to be associated with fusion.

### Orthogonal methods of fusion detection

ALK (clone D5F3) and Pan-TRK (clone EPR17341) immunohistochemistry tests were performed by NeoGenomics Laboratories (Aliso Viejo, CA). RNA-based Illumina (San Diego, California) TruSight Oncology 500 tests were performed by iSpecimen (Lexington, MA), Precision for Medicine (Frederick, MD), and Cureline (Cureline, Brisbane, CA). DNA-based GatewaySeq NGS Panel tests were performed by Washington University Pathology Services (Saint Louis, MO). Whole transcriptome RNA sequencing with STAR-Fusion analysis was performed by Azenta Life Sciences (Burlington, MA).

### Statistical analyses

95% confidence intervals for concordance were calculated using the Clopper-Pearson test (exact method) as implemented in the “binom” R library.

Pooled standard deviation of OncoPrism scores were calculated using the following formula.

$${s_{pooled}} = \sqrt {\frac{{({n_1} - 1){s_1}^2 + ({n_2} - 1){s_2}^2 + \ldots + ({n_k} - 1){s_k}^2}}{{{n_1} + {n_2} + \ldots + {n_k} - k}}}$$

where *n* and s represent the sample size and standard deviation of each group of technical replicates respectively.

95% confidence intervals of the pooled standard deviation were calculated with the following formula.

$$C{I_{lower}} = \sqrt {\frac{{(n - 1){s^2}}}{{{\chi _{\alpha /2}}^2}}}$$

$$C{I_{upper}} = \sqrt {\frac{{(n - 1){s^2}}}{{{\chi _{(1 - \alpha )/2}}^2}}}$$

n is the total number of replicates across all groups, s is the pooled standard deviation, ${X^ \wedge }2$ is the Chi squared critical value with n-1 degrees of freedom.

Significance of linear mixed effects models was determined by the likelihood ratio test. All linear mixed effects models were created in R using the “lme4” library.

## Results

### Immune checkpoint inhibitor response prediction

The workflow for the OncoPrism-HNSCC test is illustrated in Fig. 1. Our analytical validation study of OncoPrism-HNSCC ICI response prediction includes components that assess sensitivity (RNA input amount and quality), specificity (gDNA as an interferent), repeatability (intra-run variability), and intermediate precision (inter-run variability: operator, lot, day, machine) (Fig. 2A). OncoPrism scores have a possible range of 0–100, and are used to categorize patients as having low likelihood of disease control in response to ICI (1–37), indeterminate likelihood of disease control (38–51), or high likelihood of disease control (52–100) [7]. The pooled standard deviations of OncoPrism scores for the various components of the analytical validation are summarized in Table 1 and are indicative of the low variance of OncoPrism scores that might result from typical sources of variation in the laboratory.

**Table 1:** OncoPrism score pooled standard deviations for validation tests

Validation test	Number of NGS libraries in validation	Range of OncoPrism scores in validation	Pooled SD (95% CI)	Categorical concordance (95% CI)	*p* value
RNA input amount	72	22–80	1.42 (1.18–1.78)	68/72, 94% (86–98%)	< 0.001
%DV200	54	25–74	1.73 (1.46–2.14)	50/54, 93% (82–98%)	0.27
gDNA spike-in	60	27–66	1.47 (1.24–1.78)	59/60, 98% (91–100%)	< 0.001
Repeatability (intra-assay)	42	28–85	0.73 (0.60–0.94)	40/42, 95% (87–99%)	N/A
Intermediate precision (operator, lot, day)	164	27–74	0.87 (0.79–0.98)	164/164 100% (98–100%)	Operator: 0.96 Lot: 0.47 Day: 0.68
Instrument (sequencer)	42 (sequenced on each instrument)	28–74	0.77 (0.67–0.91)	42/42, 100% (92–100%)	0.24

### RNA input amount - ICI response prediction

**Fig. 3:**
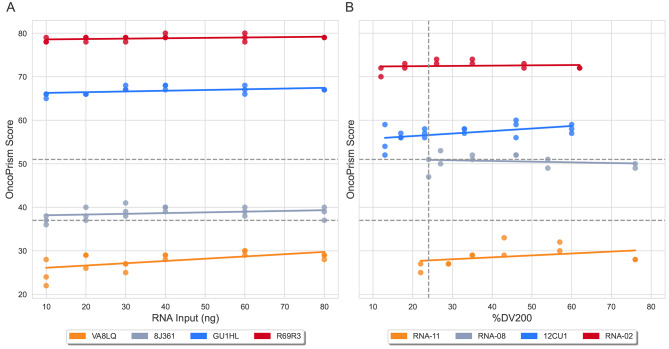
Analytical sensitivity. (**A**) Effect of varying RNA input quantity (10–80 ng) on OncoPrism scores. The nominal RNA input amount for OncoPrism-HNSCC is 40 ng. (**B**) Effect of varying %DV200 on OncoPrism scores. RNA from FFPE-preserved HNSCC RNA was subjected to Mg^2+^-mediated fragmentation for increasing times to generate samples with a range of decreasing %DV200. The dashed vertical line represents the sample RNA QC threshold for the assay (%DV200 > 24). (**A and B**) Dashed horizontal lines represent OncoPrism-HNSCC categorical thresholds. Samples with OncoPrism scores equal to or below 37 fall into the OncoPrism low group. Samples with OncoPrism scores equal to or above 52 fall into the OncoPrism high group

The nominal RNA input amount for OncoPrism-HNSCC is 40 ng, but the actual input amount can vary due to error in pipetting and RNA quantitation. Coefficients of variation for intra-batch and inter-batch RNA quantitations by multiple operators, using typical sample volumes and concentrations, were less than 6% (Supplementary Table 1). We tested 4 different RNA samples using input amounts that ranged from 10 ng to 80 ng (Fig. 3A), which exceeded the potential range of inputs that would result from expected pipetting and quantitation error. OncoPrism scores did show a slight trend of decreasing as RNA input amount decreased (Fig. 3A), and did differ significantly by RNA input amount using linear mixed effects models (Table 1). The effect size of this difference was small, however, with a pooled standard deviation of 1.42 (95%CI 1.18–1.78), for samples which had OncoPrism scores ranging from 22 to 80 (Table 1). In addition, the RNA samples spanned the range of OncoPrism groups (low, medium, high), and the call concordance across all input amounts was 94% (68 of 72, 95% CI 86–99%).

### RNA quality (%DV200) - ICI response prediction

RNA used as input for the OncoPrism-HNSCC test is derived from formaldehyde-fixed paraffin-embedded (FFPE) patient tissue. FFPE processing has detrimental effects on RNA quality that can depend on multiple factors, including delay to fixation, temperature before fixation, size and density of tissue, and time in fixative [16–18]. Accordingly, the quality of RNA extracted from FFPE-preserved tissue can be inconsistent. A commonly used measure of FFPE RNA quality is %DV200, or the percentage of RNA fragments greater than the size of 200 nt. A study by the Cancer Immune Monitoring and Analysis Centers and Cancer Immunologic Data Center Network concluded that a DV200 > 24% was a reliable QC metric for the generation of RNA-seq data [19]. Based on this finding, we set a threshold of DV200 > 24% for OncoPrism-HNSCC samples. In order to model the effect of varying %DV200 on OncoPrism scores, we subjected 4 different FFPE RNA samples to Mg^2+^/heat-mediated fragmentation for increasing times. This generated RNA samples ranging in %DV200 from 12 to 76% (Fig. 3B). Fragmented RNAs were processed in triplicate for one sample and in duplicate for the other three. OncoPrism scores for these samples did not vary significantly by %DV200 (*p* = 0.27, by linear mixed effects models). The pooled standard deviation of OncoPrism scores for these samples, which ranged from 25 to 74, was 1.73 (95%CI 1.46–2.14) (Table 1). The RNA samples spanned the range of OncoPrism groups (Fig. 3B), and the call concordance across all %DV200 levels was 93% (50 of 54, 95% CI 82–98%).

### gDNA as an interfering substance - ICI response prediction

**Fig. 4:**
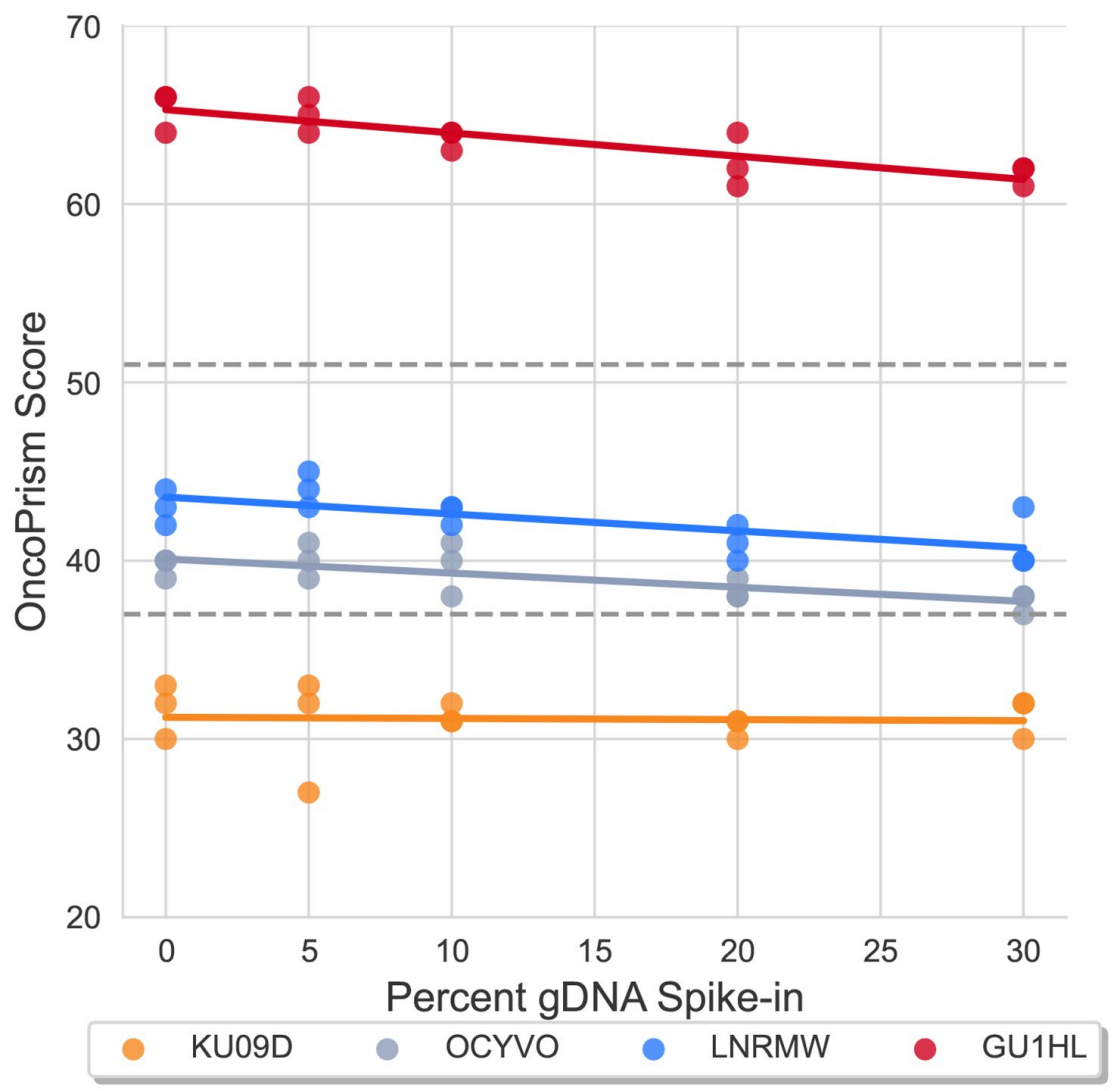
Analytical Specificity. Effect of gDNA contamination on OncoPrism score. RNA input was held steady at 40 ng and spiked with increasing percentages of gDNA by mass (0, 5, 10, 20, 30%). Dashed horizontal lines represent OncoPrism-HNSCC categorical thresholds. Samples with OncoPrism scores equal to or below 37 fall into the OncoPrism low group. Samples with OncoPrism scores equal to or above 52 fall into the OncoPrism high group

The extraction protocol used to isolate FFPE RNA for the OncoPrism-HNSCC test includes a DNase digestion. Still, some gDNA can copurify with the RNA. We therefore tested gDNA as a potential interferent of the OncoPrism-HNSCC test. DNA quantitation in a set of 65 randomly selected RNA samples indicated a mean percent DNA contamination of 12.2% (by mass), with a maximum of 25.9% (Supplementary Table 2). In order to test the effect of DNA contamination on OncoPrism scores, gDNA was spiked into RNA samples at increasing percentages by mass (0, 5, 10, 20, 30%) while RNA input was held constant at the nominal level of 40 ng (Fig. 4). OncoPrism scores did show a trend of decreasing as gDNA content increased (Fig. 4) and differed significantly by gDNA spike-in percentage using linear mixed effects models (Table 1). However, the effect size of this difference remained low, with a pooled standard deviation of 1.47 (95% CI 1.24–1.78) for OncoPrism scores that ranged from 27 to 66 (Table 1). Furthermore, OncoPrism-HNSCC categorical call concordance was high at 98% (59 of 60, 95% CI 91–100%), for samples that spanned the range of OncoPrism groups.

### Repeatability - ICI response prediction

**Fig. 5:**
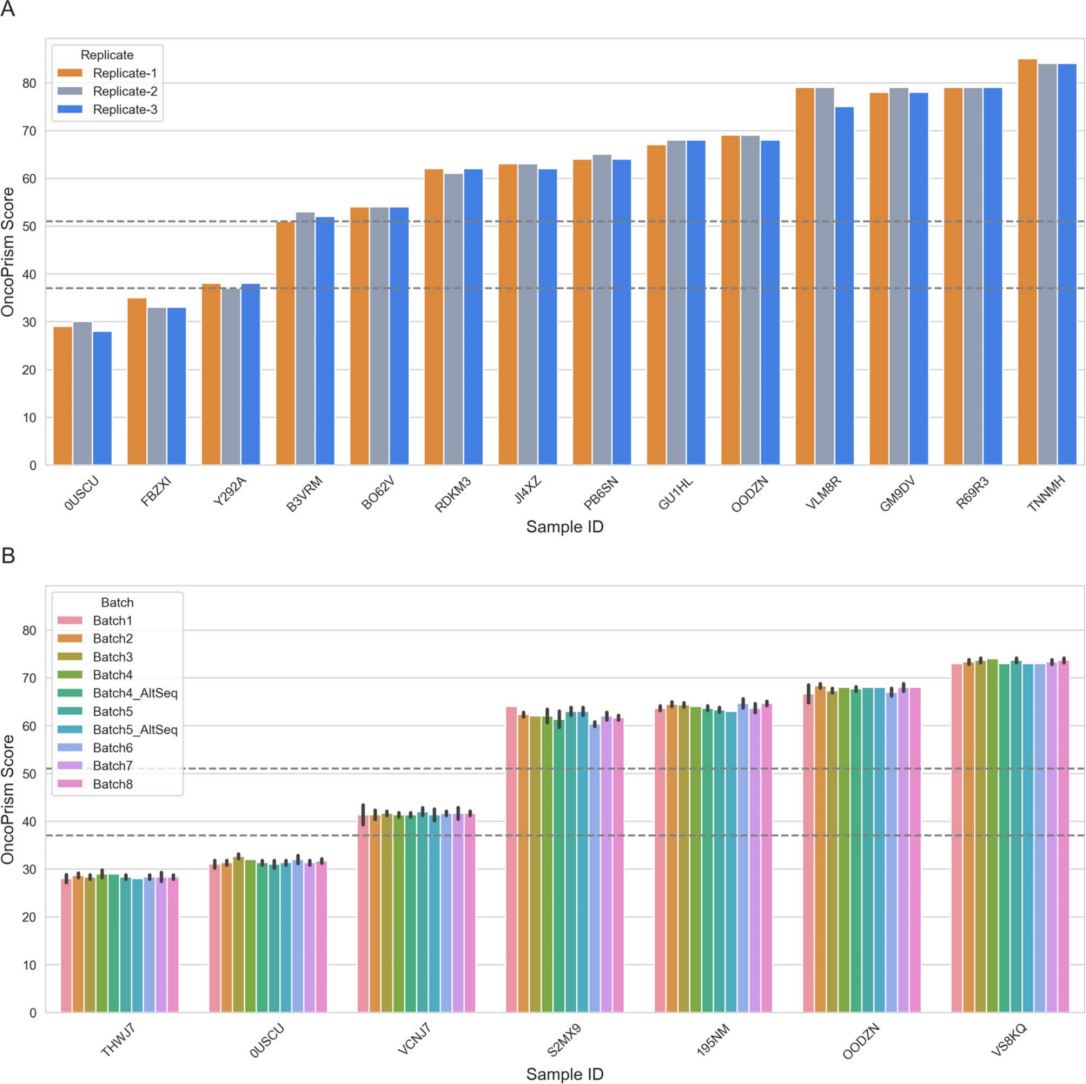
Analytical precision. (**A**) Repeatability (intra-operator variability). Fourteen different RNA samples were processed in triplicate by a single operator using the same reagent lots and instruments. (**B**) Intermediate precision (inter-operator, inter-lot, inter-day, inter-machine variability). Seven different RNA samples were processed in triplicate with multiple operators and reagent lots in 8 different batches. Bars show mean OncoPrism scores for replicates within each batch (± SD). Bars show mean OncoPrism scores for replicates within each batch (± SD). “Alt_Seq’’ indicates batches sequenced on an alternate machine. (**A and B**) Dashed horizontal lines represent OncoPrism-HNSCC categorical thresholds. Samples with OncoPrism scores equal to or below 37 fall into the OncoPrism low group. Samples with OncoPrism scores equal to or above 52 fall into the OncoPrism high group

Repeatability (intra-operator variability) for OncoPrism-HNSCC ICI prediction was assessed using 14 different clinical FFPE HNSCC samples, including those with expected OncoPrism scores that fall near decision thresholds (Fig. 5A, dashed lines). Samples were processed in triplicate by a single operator in two batches using a single reagent lot, the same instrument, and randomized sample order. Variation was low, with a pooled standard deviation of 0.73 (95% CI 0.60–0.94) for samples which had OncoPrism scores that ranged from 28 to 85 (Table 1). Categorical call concordance was 95% (60 of 63, 95% CI 87–99%).

### Intermediate precision - ICI response prediction

Intermediate precision (inter-operator, inter-lot, inter-day, inter-instrument variability) of OncoPrism-HNSCC ICI prediction was assessed with preparation of 168 NGS libraries from seven different clinical FFPE HNSCC samples with expected OncoPrism scores that spanned low, medium, and high categories (Fig. 5B). NGS libraries were prepared across eight batches with multiple operators, reagent lots (Additional-file-3_reagent-lots), and days (Table 2). Each batch consisted of triplicates of the same seven RNA samples plus controls. In addition, two batches of libraries (Batch-4, Batch-5), which were processed by different operators using different reagent lots, were sequenced on an alternate sequencer (Batch-4 Alt Seq, Batch-5 Alt Seq).

**Table 2:** Library batching scheme for intermediate precision study of ICI response prediction

Library Batch ID	Factors
Library Batch-1	Operator-1, reagent-lot-A, *Day-1
Library Batch-2	Operator-1, reagent-lot-A, Day-2
Library Batch-3	Operator-1, reagent-lot-B, Day-1
Library Batch-4	Operator-1, reagent-lot-B, Day-2
Library Batch-5	Operator-2, reagent-lot-A, Day-1
Library Batch-6	Operator-2, reagent-lot-A, Day-2
Library Batch-7	Operator-2, reagent-lot-B, Day-1
Library Batch-8	Operator-2, reagent-lot-B, Day-2

*Processing of all 8 library batches was initiated on different days. ”Day-1” and “Day-2” designate pairs of batches that differ only by start date of library processing. Specifically, Batch-1/Batch-2; Batch-3/Batch-4; Batch-5/Batch-6; Batch-7/Batch-8

Results are not reported for 4 libraries (out of 168) that failed QC. Overall, the categorical call concordance was 100% (164 of 164, 95% CI 98–100%) (Table 1). The pooled standard deviation for all batches was 0.87 (95% CI 0.79–0.98), which had OncoPrism scores that ranged from 27 to 74 (Table 1). For sequencer comparison (Batch-4, Batch-5, Batch-4 Alt Seq, Batch-5 Alt Seq), the pooled standard deviation was 0.77 (95% CI 0.67–0.9), with OncoPrism scores that ranged from 28 to 74 (Table 1). Categorical call concordance for sequencer comparison was 100% (95% CI 91.6–100%). Linear mixed-effects models indicated that OncoPrism scores did not differ significantly for all factors tested overall (Table 1, Supplementary Table 3), or for any of the separate categorical factors tested (Table 1, Supplementary Table 3).

.

### *NTRK* and *ALK* gene fusion detection

In addition to providing guidance for ICI treatment decisions, OncoPrism-HNSCC tests for the presence of rearrangements in *NTRK1/2/3* and *ALK* genes. Fusion status of these genes is not calculated into the OncoPrism ICI prediction score, but rather informs treatment options for those rare patients whose tumors possess them and could potentially benefit significantly from available non-ICI targeted therapies. Results for the analytical validation of the fusion-detection component of OncoPrism-HNSCC are summarized in Table 3. The validations include repeatability (intra-run variability) intermediate precision (inter-run variability: operator, lot, day, instrument), sensitivity (RNA input amount, lower limit of detection), specificity (gDNA as an interferent), and comparison with orthogonal methods of fusion detection (Fig. 2B).

**Table 3:** Summary of fusion-gene detection validation results

	Factors tested	Sample numbers	Expected fusions	Concordance
Repeatability (intra-run)	Single operator, single reagent lot, single sequencer	3 different clinical RNA samples (2 fusion-positive, 1 fusion-negative) in duplicate	*EML4::NTRK3*, *EML4::ALK*, None	100% (6/6, 95% CI 54.1–100%)
Intermediate precision (operator, lot, day)	3 operators, multiple reagent lots, multiple days	5 different clinical RNA samples (1 fusion-negative, 4 fusion-positive)	*EML4::NTRK3*, *EML4::ALK*, *TPM3::NTRK1*, *ETV6::NTRK3*, None	100% (18/18, 95% CI 81.5–100%)
Intermediate precision (instrument)	2 different sequencers	10 different clinical sample, RNAs, fusion-positive and fusion-negative reference RNAs	*EML4::NTRK3* *NPM1::ALK* *TPM3::NTRK1* *EML4::ALK* *QKI::NTRK2* *ETV6::NTRK3* *None*	100% (12/12, 95% CI 73.5–100%)
Sensitivity (RNA input amount)	Nominal RNA input amount is 50 ng. Input amounts tested were 20, 35, 50, 65, 80 ng	3 different fusion-positive clinical RNA samples	*TPM3::NTRK1* *ETV6::NTRK3* *EML4::ALK*	100% (15/15, 95% CI 78.2–100%)
Sensitivity (lower limit of detection)	Fusion-positive RNA samples mixed with fusion-negative reference RNA. Final fusion-positive RNA percentages in mixes: 1, 5, 10, 25, 100%	2 different fusion-positive RNA clinical samples and fusion-positive reference RNA	*TPM3::NTRK1* *EML4::ALK* *QKI::NTRK2* *ETV6::NTRK3*	Overall, at level of fusion genes detected: 86.7% (26/30, 95% CI 69.3–96.2%)
Specificity (gDNA as an interferent)	Clinical sample RNA input held constant with increasing gDNA spike-in percentages at: 0, 5, 10, 20, 30%	2 different fusion-positive and 1 fusion-negative RNA clinical samples	*EML4::NTRK3* *EML4::ALK*	100% (15/15, 95% CI 78.2–100%)
Clinical samples	Orthogonal validation of clinical samples, with at least 1 of the following tests: ALK IHC, Pan-Trk IHC, TSO500 (RNA), GatewaySeq (DNA), RNA-seq with STAR-Fusion	67 clinical samples, including 55 Head and Neck, 8 Lung, 2 Colorectal, 2 NHL; includes 14 with known fusion-positive status	*EML4::ALK* *TPM3::NTRK1* *EML4::NTRK3* *NPM1::ALK* *CLTC::ALK* *ETV6:NTRK3* None	100% (67/67, 95% CI 94.6–100%)
Fusion-positive reference RNA	Process controls	15 batches	*TPM3::NTRK1* *EML4::ALK* *QKI::NTRK2* *ETV6::NTRK3*	Overall, at level of fusion genes detected: 100% (60/60, 95% CI 94–100%)
Fusion-negative reference RNA	Process controls	15 batches	None	100% (0 of 0)

### Validation samples - fusion detection

We acquired 14 different FFPE tumor samples where prior analysis had indicated that *ALK* or *NTRK* fusion status was positive (Supplementary Table 4). This included 8 lung, 2 colorectal, 2 non-Hodgkin’s Lymphoma (NHL), and 2 head and neck. An additional 53 fusion-negative head and neck cases were used. We also used fusion-positive reference RNA (Horizon Discovery) with validated rearrangements in *NTRK1/2/3* and *ALK* genes, as well as fusion-negative reference RNA (Horizon Discovery) with validated absence of rearrangements in *NTRK1/2/3* and *ALK* genes.

### Repeatability (intra-run) - fusion detection

Three different FFPE RNA samples were processed as replicates in the same batch, using the same reagent lots and instruments. Two of the samples were known fusion-positive and one was fusion-negative (Supplementary Table 5). Fusion detection concordance was 100% (6/6, 95% CI 54.1–100%) across all replicates (Table 3, Supplementary Table 5).

### Intermediate precision - fusion detection

RNA from one fusion-negative and four different fusion-positive FFPE RNA samples were processed as replicates in separate batches. Samples were repeated by both the same operator and by different operators using several reagent lots (Additional-file-3_reagent-lots). Fusion detection concordance was 100% (18/18, 95% CI 81.5–100%) across all replicates (Table 3, Supplementary Table 6). In addition, libraries for ten clinical samples, fusion-positive reference RNA, and fusion-negative reference RNA, were re-sequenced on a different sequencer to test comparability of different machines. Fusion detection for all twelve samples (5 fusion-positive, 7 fusion-negative) was 100% concordant (12/12, 95% CI 73.5–100%) between different sequencers (Table 3, Supplementary Table 7).

### Sensitivity (RNA input amount) - fusion detection

The nominal RNA input amount for OncoPrism-HNSCC fusion-detection is 50 ng. Inputs ranging from 20 ng to 80 ng were used to test the sensitivity of the assay to varying RNA input amounts. Three different fusion-positive clinical RNA samples were tested using 5 different input amounts: 20, 35, 50, 65, 80 ng. There was 100% concordance (15/15, 95% CI 78.2–100%) with expected fusions detected across all RNA input levels (Table 3, Supplementary Table 8).

### Sensitivity (lower limit of detection) - fusion detection

The minimum tumor cellularity for OncoPrism-HNSCC FFPE samples is 10%. In order to model the effect of decreasing tumor cellularity on fusion gene detection, fusion-positive RNA samples were combined with fusion-negative reference RNA at varying ratios, by mass. The fusion-positive RNAs used to prepare these mixes included two different clinical samples and fusion-positive reference RNA. The final percentages of fusion-positive RNA in the mixes were as follows: 100%, 25%, 10%, 5%, and 1%. Overall concordance of fusion-gene detection in the mixes was 86.7% (26 of 30, 95% CI 69.3–96.2%, Table 3, Supplementary Table 9), which accounts for the presence of 3 different fusion genes in the fusion-positive reference RNA. All fusion genes were detected in all mixes consisting of 10% or greater fusion-positive RNA (Supplementary Table 9).

The gene fusion in clinical sample PB05R was detected down to the 5%–positive-reference–mix (Supplementary Table 9). FFPE clinical sample PB05R had a tumor cellularity of 25% (Supplementary Table 4), which corresponds to an effective tumor cellularity of ∼ 1% for the 5%–PB05R–mix. Similarly, the gene fusion in clinical sample UWD23 was detected down to the 10%–positive-reference–mix. FFPE clinical sample UWD23 had a tumor cellularity of 60% (Supplementary Table 4), which corresponds to an effective tumor cellularity of ∼ 6% for the 10%–UWD23–mix. In both cases, the gene fusions were detected below an effective 10% minimum tumor cellularity, which is the predetermined threshold for OncoPrism-HNSCC test. Although the titrations with fusion-negative reference RNA do not directly correspond to decreasing levels of tumor cellularity, this experiment helps guide the lower limit of detection for this test.

In addition to tumor cellularity, the level of fusion transcript expression affects the sensitivity of fusion detection. Copy numbers of *ALK* and *NTRK1/2/3* fusion transcripts present in the fusion-positive reference RNA were quantitated by the manufacturer using digital droplet PCR. We extrapolated the fusion-transcript copy number input into NGS library preparations of the fusion-positive/fusion-negative reference RNA mixes we prepared (Supplementary Table 10). The fusion gene detected with the lowest copy number input was *EML4::ALK* in the 1% fusion-positive/fusion-negative reference RNA mix, at 58 copies (Supplementary Table 10). Considering that the estimated mRNA content of mammalian cells is between 50,000 and 300,000 transcripts per cell [20], the detection of *EML4::ALK* with an input of 58 copies indicates that OncoPrism-HNSCC fusion detection is highly sensitive.

### Specificity (gDNA as an interferent) - fusion detection

OncoPrism-HNSCC uses RNA to detect gene fusions. As described above for the validation of ICI prediction, co-purifying gDNA is a potential interferent of the OncoPrism-HNSCC test. We therefore tested the effect of gDNA spike-ins on fusion gene detection. Three different FFPE RNA samples were used, two of which were known to be fusion-positive and one of which was fusion-negative (Supplementary Table 11). Genomic DNA was extracted from additional FFPE tissue sections from the same samples used for RNA extraction. RNA input was held constant at 50 ng. Same-sample gDNA was spiked into each RNA at increasing percentages before running the OncoPrism-HNSCC fusion detection test. The spike-in percentages were 0, 5, 10, 20, and 30% (by mass). Categorical call concordance was 100% (15 of 15, 95% CI 78.2–100%) across all levels of gDNA tested (Table 3, Supplementary Table 12). Increasing gDNA contamination did not result in any false positive or false negative fusion detections.

### Fusion detection in clinical FFPE RNA samples using orthogonal methods

A cohort of 67 FFPE clinical samples was assessed for fusion gene status using OncoPrism-HNSCC (Supplementary Table 13). These samples were subjected to testing by independent labs using orthogonal methods for fusion gene detection (see Methods). Fourteen of the 67 clinical samples were known to be fusion positive for *ALK* or *NTRK1/3* genes (Supplementary Table 4). The other 53 were head and neck cases with no prior knowledge of fusion gene status (Supplementary Table 13). We were unable to acquire clinical samples with known *NTRK2* gene rearrangement. *NTRK2* fusions are rare across nearly all cancers, with an overall prevalence of just 0.05% [21]. However, our fusion-positive reference RNA includes gene fusions for all 4 OncoPrism-HNSCC reported genes, including *NTRK2*. The *NTRK2* fusion was detected across all 15 positive control samples processed as batch controls for technical assessments (Table 3), and in all fusion-positive/fusion-negative reference RNA mixes used for the sensitivity experiments (Supplementary Table 9).

Overall, for the clinical FFPE samples, call concordance between OncoPrism-HNSCC and orthogonal methods for fusion gene detection was 100% (67 of 67; 95% CI 94.6–100%) (Table 3, Supplementary Table 13). Performance metrics for accuracy, negative percent agreement, and positive percent agreement for OncoPrism-HNSCC fusion detection compared to orthogonal methods, along with 95% confidence intervals, are shown in Supplementary Table 14.

## Discussion

### OncoPrism-HNSCC ICI response prediction

OncoPrism-HNSCC is a laboratory developed test that predicts disease control rate and progression free survival in response to anti-PD-1 therapy in pre-treatment RM-HNSCC patients. The test was clinically validated in two separate cohorts of patient samples from 17 clinical sites from across the United States [7]. PD-L1 CPS is widely used to predict response to PD-1 inhibitors across many cancer types. Unfortunately, PD-L1 CPS has poor accuracy in HNSCC and many other indications due to its poor specificity [5, 22]. Here, we report the analytical validation of OncoPrism-HNSCC, showing the test’s robustness and tolerance of potential sources of variation. The low analytical variance allows for the reliable detection of biological signals above noise found in RNA sequencing data and was important for the development and training of the OncoPrism-HNSCC biomarker model, and ultimately the successful clinical validation of the test [7]. The low variance in OncoPrism-HNSCC scoring ultimately translates to low variance of categorical calls into low, medium, and high patient response groups. This confidence in categorization is key when doctors choose which treatment to prioritize, such as chemotherapy, chemotherapy combined with immune checkpoint inhibitors, or immune checkpoint inhibitor monotherapy.

The CDC’s Analytic and Clinical validity, Clinical utility and associated Ethical, legal and social implications Project (ACCE) was established to ensure evidence-based evaluation of genomics-based clinical tests [14, 23]. Consistent with the ACCE model, our analytical validation study includes evaluation of the test’s sensitivity to RNA input quantity and quality, specificity in the presence of gDNA as a potential interferent, repeatability of technical replicates by a single user, and intermediate precision when processing samples across multiple operators, reagent lots, days, and sequencers.

OncoPrism-HNSCC was robust across a range of RNA inputs. The nominal RNA input amount for OncoPrism-HNSCC ICI prediction is 40 ng total RNA. We tested four different FFPE RNA samples at a range of inputs from 10 to 80 ng, a level down to four-fold below the nominal input. While there was a significant trend toward lower OncoPrism scores with lower RNA input, the effect size was small (Table 1).

Decreasing %DV200 did not significantly impact OncoPrism scores. We modeled the effect RNA degradation seen in FFPE tissue, which largely results from the activity of endogenous RNases [24], by subjecting FFPE sample RNA to divalent cation mediated fragmentation (see Methods). Across six %DV200 values for each of the four different FFPE RNA samples, there was no significant trend of change in OncoPrism scores, regardless of whether libraries failing QC were included in the analysis (Table 1).

It is not uncommon for genomic DNA to co-purify with RNA extracted from FFPE tissue. Among 65 randomly selected validation samples, the median level of contaminating gDNA was 12% (as a percent of the mass of RNA), with a maximum of 26% (Supplementary Table 2). To test the effect of gDNA on OncoPrism-HNSCC results, we spiked gDNA into RNA samples at increasing concentrations, up to 30% (Fig. 4). Increasing gDNA did correlate with decreasing OncoPrism scores (*p* < .001) (Fig. 4). However, the effect size was small, with a pooled standard deviation of 1.43 (95% CI 1.24–1.78), representing just 3.7% of the range of scores in the gDNA spike-in study (Table 1). In addition, the categorical call concordance of samples spiked with gDNA remained high (98.3%, 59/60, 95% CI 91.1–100%). Ongoing work is investigating strategies for further reducing gDNA contamination and additional gDNA-related QC metrics.

OncoPrism-HNSCC had low technical variability within and across multiple batches, operators, reagent lots, and instruments. To measure repeatability within a single batch, 14 different RNA patient samples were processed in triplicate by a single operator, including four samples with OncoPrism scores close to a decision threshold (Fig. 5A). Despite this proximity to decision thresholds, categorical call concordance for these four samples was 83.3% (10/12, 95% CI 52–98%), and overall categorical call concordance for all samples was 95.2% (40/42, 86.7–99.0%). The pooled standard deviation for this set of samples was 0.73 (95% CI 0.60–0.94), representing just 1.3% of the range of scores in the repeatability study (28–85). Likewise, the variance due to multiple operators running the test in multiple batches using multiple kit lots and machines was represented by just 1.9% of the range of scores in the intermediate precision study (27–74), or just 0.87% of the entire score range for the test (0–100), and none of these factors were significantly correlated with score (Table 1).

OncoPrism-HNSCC’s analytical variance compares favorably to similar intermediate precision studies from other RNA-based tests. For example, the Percepta GSC test for the cancer risk assessment of suspicious lung nodules shows a variance that represents 3.9% of the entire score range of the test in their intermediate precision study [25]. The Afirma GSC test for classifying benign vs. malignant thyroid nodules shows a variance that represents 3.4% of the entire score range of the test [26]. The Prosigna assay for the assessment of the risk of recurrence of breast cancer shows a variance of 0.6–0.8% of the range of possible scores (0–100) for the test [27]. Thus, the analytical variance of the OncoPrism-HNSCC ICI response prediction test is of a similar magnitude as other established RNA-based tests in current clinical use.

### OncoPrism-HNSCC Gene Fusion Detection

While *NTRK* and *ALK* gene fusions are rare across most solid tumor types, multiple studies have demonstrated that patients whose tumors are positive for these fusions can benefit significantly from therapies that target the aberrant activities resulting from the alteration of the receptors encoded by these genes [11, 28]. We use the FFPE patient RNA extracted to run the OncoPrism-HNSCC ICI response prediction test as input to additionally test for the presence of *NTRK1/2/3* and *ALK* gene fusions.

OncoPrism-HNSCC detects *NTRK* and *ALK* gene fusions, agnostic of fusion partner, with high sensitivity and specificity. The high sensitivity of OncoPrism-HNSCC is borne out by limit of detection studies where *EML4::ALK* rearrangements were identified in fusion-positive/fusion-negative RNA mixes down to an estimated level of 58 copies of fusion transcript in the 50 ng RNA input of the assay (Supplemental Table 10). Validation studies of RNA input amount, gDNA spike-ins, repeatability (intra-assay), and intermediate precision (multiple operators, reagent lots, days, sequencers), yielded 100% concordances for fusion detection (Table 3).

Importantly, OncoPrism-HNSCC fusion detection in 67 FFPE clinical samples was compared to orthogonal methods of fusion detection carried out by independent labs (Supplemental Table 13). The 67 samples included 14 with known *NTRK* or *ALK* gene fusions, and an additional 53 of unknown fusion gene status (Supplemental Table 4, Supplemental Table 13). The orthogonal methods for fusion detection included one or more of the following: TruSight Oncology 500 (RNA-based NGS), GatewaySeq (DNA-based NGS), whole transcriptome RNA-seq with STAR-Fusion, Pan-TRK IHC (clone EPR17341), and ALK IHC (clone D5F3) (Supplemental Table 13). Multiple orthogonal tests for fusion detection were performed for many cases (e.g., IHC and NGS). For six samples, the orthogonal assay results were not concordant with each other. For the purposes of assessing concordance with OncoPrism-HNSCC, the “expected” orthogonal fusion-detection call in these cases was the result reported by the majority of orthogonal tests, if applicable (Supplemental Table 13). In two cases the RNA-based TSO500 reported a gene fusion but the DNA based GatewaySeq did not, and IHC testing was unavailable. In these cases, the TSO500 result was used for determining concordance, due to the generally higher sensitivity of RNA-based versus DNA-based fusion detection [29]. Overall, OncoPrism-HNSCC was 100% concordant with orthogonal methods of fusion detection for the 67 FFPE clinical samples tested (Supplemental Table 13). Together with the 100% concordant results for fusion detection studies of RNA input amount, gDNA as an interferent, repeatability, and intermediate precision, we have demonstrated the robustness and reliability of OncoPrism-HNSCC fusion detection.

## Study limitations

Variations in some preanalytical factors that might affect sample quality were not assessed in this study. This includes age and storage conditions of FFPE blocks, sample stability of extracted RNA, and tissue fixation protocols practiced by different histology labs. While the %DV200 analysis included here (Fig. 3B) models RNA fragmentation that might result from varying warm ischemic times and fixation, it does not model potential chemical modification of RNA due to fixatives.

Tumor heterogeneity is a long-standing challenge for biomarker testing, and can arise from the genomic instability of cancer cells that gives rise to intra- and inter-tumor subpopulations of differing phenotypes. Tumors also can have non-uniform distributions of immune, stromal and endothelial cells. While OnocoPrism-HNSCC is performed using bulk RNA-seq on FFPE sections, this study did not assess the effect of sampling biopsies from different regions of the same tumor, or different tumors from the same patient.

Another limitation of the study is that inter-operator variation was formally tested with only two operators. As OncoPrism-HNSCC gains more usage, it will be important to test inter-operator variation more robustly. We note that four operators participated in end-to-end preparation of NGS libraries for ICI response prediction and demonstrated low intra-batch variation of OncoPrism scores, as represented by the data herein.

## Conclusions

The OncoPrism-HNSCC assay demonstrates robust and reliable analytical performance, laying a solid foundation for its clinical use despite the variability common in clinical samples and laboratory settings. Varying RNA inputs as low as four-fold below the nominal amount, and RNA quality levels below the sample QC threshold, had little or no significant impact on ICI response prediction scores. Genomic DNA spike-ins up to 30% resulted in only minor score variations. The overall variance, measured across multiple operators, reagent lots, batches, and sequencers, was minimal. Additionally, *NTRK* and *ALK* gene fusion detection showed 100% concordance with orthogonal methods. These results validate the assay’s sensitivity, specificity, and precision, ensuring its reliability for predicting ICI response and identifying rare oncogenic rearrangements in RM-HNSCC patients.

## Electronic supplementary material

Below is the link to the electronic supplementary material.

Supplementary File 1: Supplementary Table 1 to Supplementary Table 14

Additional file 1: Includes a key to which patient samples were used for the different analyses

Additional file 2: Includes reagent part and lot numbers used for the reproducibility study

Additional file 3: Includes available patient demographic data

## Data Availability

The datasets generated and/or analysed during the current study are available in the Gene Expression Omnibus (GEO) repository, GSE270047, or are included in this published article and supplementary material.

## References

[1] Johnson DE, Burtness B, Leemans CR, Lui VWY, Bauman JE, Grandis JR. Head and neck squamous cell carcinoma. Nat Reviews Disease Primers. 2020;6. 10.1038/s41572-020-00224-3 PMC7944998 33243986

[2] Park JC, Krishnakumar HN, Saladi SV. Current and future biomarkers for Immune checkpoint inhibitors in Head and Neck squamous cell carcinoma. Curr Oncol. 2022;29:4185–98. 35735443 10.3390/curroncol29060334 PMC9221564

[3] Cohen EEW, Soulières D, Le Tourneau C, Dinis J, Licitra L, Ahn M-J, et al. Pembrolizumab versus methotrexate, docetaxel, or cetuximab for recurrent or metastatic head-and-neck squamous cell carcinoma (KEYNOTE-040): a randomised, open-label, phase 3 study. Lancet. 2019;393:156–67. 30509740 10.1016/S0140-6736(18)31999-8

[4] Yilmaz E, Ismaila N, Bauman JE, Dabney R, Gan G, Jordan, Richard, et al. Immunotherapy and Biomarker Testing in Recurrent and Metastatic Head and Neck cancers: ASCO Guideline. J Clin Oncol. 2022;41:1132–46. 36521102 10.1200/JCO.22.02328

[5] Burtness B, Harrington KJ, Greil R, Soulières D, Tahara M, de Castro G, et al. Pembrolizumab alone or with chemotherapy versus cetuximab with chemotherapy for recurrent or metastatic squamous cell carcinoma of the head and neck (KEYNOTE-048): a randomised, open-label, phase 3 study. Lancet. 2019;394:1915–28. 31679945 10.1016/S0140-6736(19)32591-7

[6] Harrington KJ, Burtness B, Greil ; Richard, Soulì D, Tahara M et al. Gilberto De Castro ;,. Pembrolizumab With or Without Chemotherapy in Recurrent or Metastatic Head and Neck Squamous Cell Carcinoma: Updated Results of the Phase III KEYNOTE-048 Study. J Clin Oncol. 2022;41:790–802. 10.1200/JCO.21.02508 PMC9902012 36219809

[7] Flanagan KC, Earls J, Hiken J, Wellinghoff RL, Ponder MM, Mcleod HL, et al. Multicenter validation of an RNA-based assay to predict anti-PD-1 disease control in patients with recurrent or metastatic head and neck squamous cell carcinoma: the PREDAPT study. J Immunother Cancer. 2024;12:e009573. 39489541 10.1136/jitc-2024-009573 PMC11535711

[8] Flanagan KC, Earls J, Schillebeeckx I, Hiken J, Wellinghoff RL, LaFranzo NA, et al. Multidimensional biomarker predicts disease control in response to immunotherapy in recurrent or metastatic head and neck squamous-cell carcinoma. J Cancer Res Clin Oncol. 2023;149:14125–36. 37552307 10.1007/s00432-023-05205-z PMC10590294

[9] Yoshino T, Pentheroudakis G, Mishima S, Overman MJ, Yeh KH, Baba E, et al. JSCO—ESMO—ASCO—JSMO—TOS: international expert consensus recommendations for tumour-agnostic treatments in patients with solid tumours with microsatellite instability or NTRK fusions. Ann Oncol. 2020;31:861–72. 32272210 10.1016/j.annonc.2020.03.299

[10] Cocco E, Scaltriti M, Drilon A. NTRK fusion-positive cancers and TRK inhibitor therapy. Nat Reviews Clin Oncol. 2018;15:731–47. 10.1038/s41571-018-0113-0 PMC6419506 30333516

[11] Shreenivas A, Janku F, Gouda MA, Chen HZ, George B, Kato S et al. ALK fusions in the pan-cancer setting: another tumor-agnostic target? Npj Precision Oncol. 2023;7. 10.1038/s41698-023-00449-x PMC10542332 37773318

[12] Westphalen CB, Krebs MG, Le Tourneau C, Sokol ES, Maund SL, Wilson TR et al. Genomic context of NTRK1/2/3 fusion-positive tumours from a large real-world population. NPJ Precis Oncol. 2021;5. 10.1038/s41698-021-00206-y PMC8292342 34285332

[13] Li MM, Datto M, Duncavage EJ, Kulkarni S, Lindeman NI, Roy S, et al. Standards and guidelines for the interpretation and reporting of sequence variants in Cancer: a Joint Consensus Recommendation of the Association for Molecular Pathology, American Society of Clinical Oncology, and College of American Pathologists. J Mol Diagn. 2017;19:4–23. 27993330 10.1016/j.jmoldx.2016.10.002 PMC5707196

[14] Haddow J, Palomaki G. ACCE: a model process for evaluating data on emerging genetic tests. New York: Oxford University Press; 2003.

[15] Schillebeeckx I, Armstrong JR, Forys JT, Hiken J, Earls J, Flanagan KC, et al. Analytical Performance of an Immunoprofiling Assay based on RNA models. J Mol Diagn. 2020;22:555–70. 32036085 10.1016/j.jmoldx.2020.01.009

[16] Jones W, Greytak S, Odeh H, Guan P, Powers J, Bavarva J et al. Deleterious effects of formalin-fixation and delays to fixation on RNA and miRNA-Seq profiles. Sci Rep. 2019;9. 10.1038/s41598-019-43282-8 PMC6502812 31061401

[17] Chung JY, Braunschweig T, Williams R, Guerrero N, Hoffmann KM, Kwon M, et al. Factors in tissue handling and processing that impact RNA obtained from formalin-fixed, paraffin-embedded tissue. J Histochem Cytochem. 2008;56:1033–42. 18711211 10.1369/jhc.2008.951863 PMC2569903

[18] von Ahlfen S, Missel A, Bendrat K, Schlumpberger M. Determinants of RNA quality from FFPE samples. PLoS ONE. 2007;2. 10.1371/journal.pone.0001261 PMC2092395 18060057

[19] Zeng Z, Fu J, Cibulskis C, Jhaveri A, Gumbs C, Das B, et al. Cross-site concordance evaluation of tumor DNA and RNA sequencing platforms for the CIMAC-CIDC network. Clin Cancer Res. 2021;27:5049–61. 33323402 10.1158/1078-0432.CCR-20-3251 PMC8203757

[20] Marinov GK, Williams BA, McCue K, Schroth GP, Gertz J, Myers RM, et al. From single-cell to cell-pool transcriptomes: stochasticity in gene expression and RNA splicing. Genome Res. 2014;24:496–510. 24299736 10.1101/gr.161034.113 PMC3941114

[21] Sweeney SM, Cerami E, Baras A, Pugh TJ, Schultz N, Stricker T, et al. AACR project genie: powering precision medicine through an international consortium. Cancer Discov. 2017;7:818–31. 28572459 10.1158/2159-8290.CD-17-0151 PMC5611790

[22] Lu S, Stein JE, Rimm DL, Wang DW, Bell JM, Johnson DB, et al. Comparison of Biomarker modalities for Predicting response to PD-1/PD-L1 checkpoint blockade: a systematic review and Meta-analysis. JAMA Oncol. 2019;5:1195–204. 31318407 10.1001/jamaoncol.2019.1549 PMC6646995

[23] Teutsch SM, Bradley LA, Palomaki GE, Haddow JE, Piper M, Calonge N, et al. The evaluation of genomic applications in practice and prevention (EGAPP) initiative: methods of the EGAPP working group. Genet Sci. 2009;11:3–14. 10.1097/GIM.0b013e318184137c PMC2743609 18813139

[24] Bussolati G, Annaratone L, Medico E, D’Armento G, Sapino A. Formalin fixation at low temperature better preserves nucleic acid integrity. PLoS ONE. 2011;6. 10.1371/journal.pone.0021043 PMC3115967 21698245

[25] Johnson MK, Wu S, Pankratz DG, Fedorowicz G, Anderson J, Ding J et al. Analytical validation of the Percepta genomic sequencing classifier; an RNA next generation sequencing assay for the assessment of Lung Cancer risk of suspicious pulmonary nodules. BMC Cancer. 2021;21. 10.1186/s12885-021-08130-x PMC8045183 33849470

[26] Hao Y, Choi Y, Babiarz JE, Kloos RT, Kennedy GC, Huang J et al. Analytical verification performance of Afirma genomic sequencing classifier in the diagnosis of cytologically indeterminate thyroid nodules. Front Endocrinol (Lausanne). 2019;10 JULY. 10.3389/fendo.2019.00438 PMC6620518 31333584

[27] Nielsen T, Wallden B, Schaper C, Ferree S, Liu S, Gao D et al. Analytical validation of the PAM50-based Prosigna Breast Cancer Prognostic Gene Signature Assay and nCounter analysis system using formalin-fixed paraffin-embedded breast tumor specimens. BMC Cancer. 2014;14. 10.1186/1471-2407-14-177 PMC4008304 24625003

[28] Lassen U, Bokemeyer C, Garcia-Foncillas J, Italiano A, Vassal G, Paracha N, et al. Prognostic Value of Neurotrophic Tyrosine Receptor Kinase Gene Fusions in Solid Tumors for overall survival: a systematic review and Meta-analysis. JCO Precis Oncol. 2023. 10.1200/po.22.00651 37384865 10.1200/PO.22.00651 PMC10581655

[29] Heyer EE, Deveson IW, Wooi D, Selinger CI, Lyons RJ, Hayes VM et al. Diagnosis of fusion genes using targeted RNA sequencing. Nat Commun. 2019;10. 10.1038/s41467-019-09374-9 PMC6437215 30918253

