# Supplementary material for: Analytical performance of OncoPrism-HNSCC, an RNA-based assay to inform immune checkpoint inhibitor treatment decisions for recurrent/metastatic head and neck squamous cell carcinoma
Source: BMC Cancer. 2025 Jan 7;25:21. doi: 10.1186/s12885-024-13362-8 (PMC11705923; doi:10.1186/s12885-024-13362-8)
Supplement: Supplementary file 1 — Supplementary Table 1 to Supplementary Table 14 [file 12885_2024_13362_MOESM1_ESM.docx]

**Supplementary Table 1.** %CVs for RNA dilutions and concentration assays across 4 operators

|  | **Operator-1** | **Operator-2** | **Operator-3** | **Operator-4** | **All operators** |
| --- | --- | --- | --- | --- | --- |
| Replicate 1 | 2.51 | 2.6 | 2.56 | 2.68 | NA |
| Replicate 2 | 2.7 | 2.57 | 2.40 | 2.77 | NA |
| Replicate 3 | 2.52 | 2.54 | 2.40 | 2.75 | NA |
| Replicate 4 | 2.55 | 2.53 | 2.30 | NA | NA |
| Replicate 5 | 2.5 | 2.58 | 2.30 | NA | NA |
| mean | 2.56 | 2.56 | 2.39 | 2.73 | 2.54 |
| ST DEV | 0.08 | 0.03 | 0.11 | 0.05 | 0.13 |
| %CV | 3.23 | 1.12 | 4.45 | 1.73 | 5.28 |

Five-fold dilutions of Qubit RNA HS Standard #2 were prepared by each of four different operators. Replicates of 2 µl aliquots were assayed according to manufacturer’s instructions. Measurements are shown in ng/µl.

**Supplementary Table 2** Descriptive statistics for percent DNA contamination in 65 RNA samples

| Mean | 12.2 |
| --- | --- |
| Median | 11.8 |
| Standard Deviation | 5.2 |
| Minimum | 2.4 |
| Maximum | 25.9 |
| Count | 65 |

**Supplementary Table 3** OncoPrism Score significance testing for intermediate precision

| **Factor tested** | ***p* value (linear mixed effects models)** |
| --- | --- |
| Operator | 0.96 |
| Reagent lot | 0.47 |
| Day (Batch-1 *vs* Batch-2) | 0.22 |
| Day (Batch-3 *vs* Batch-4) | 0.27 |
| Day (Batch-5 *vs* Batch-6) | 0.81 |
| Day (Batch-7 *vs* Batch-8) | 0.43 |
| Day (overall) | 0.68 |
| Machine (alternate sequencer) | 0.24 |

**Supplementary Table 4** Fusion-positive clinical samples

| **Sample ID** | **Cancer Indication** | **Expected Fusion** | **Tumor Cellularity** |
| --- | --- | --- | --- |
| UWD23 | Lung | *EML4::ALK* | 60% |
| 6MV24 | NSCLC | *EML4::ALK* | 40% |
| 73Z6P | NSCLC | *EML4::ALK* | 30% |
| COUCS | NSCLC | *EML4::ALK* | 30% |
| O2USE | NSCLC | *EML4::ALK* | 30% |
| ST6HK | NSCLC | *EML4::ALK* | 60% |
| U6DI7 | NSCLC | *EML4::ALK* | 40% |
| WM11J | NSCLC | *EML4::ALK* | 70% |
| B1NVL | Colorectal | *TMP3::NTRK1* | 40% |
| RNMWE | Colon | *EML4::NTRK3* | 80% |
| 1KS6A | NHL (Anaplastic Large Cell Lymphoma) | *NPM1::ALK* | 90% |
| JRL2T | NHL (Diffuse Large B-cell Lymphoma) | *CLTC::ALK* | 70% |
| PB05R | Head and Neck (salivary gland) | *ETV6:NTRK3* | 25% |
| KXFRY | Head and Neck (thyroid) | *ETV6:NTRK3* | 30% |

**Supplementary Table 5** Fusion detection - repeatability (intra-run)

| **Sample ID** | **Expected Fusion Result** | **Observed Fusion Result** | **Concordant?** |
| --- | --- | --- | --- |
| RNMWE_Rep1 | *EML4::NTRK3* | *EML4::NTRK3* | Yes |
| RNMWE_Rep2 | *EML4::NTRK3* | *EML4::NTRK3* | Yes |
| UWD23_Rep1 | *EML4::ALK* | *EML4::ALK* | Yes |
| UWD23_Rep2 | *EML4::ALK* | *EML4::ALK* | Yes |
| MR5VU_Rep1 | not detected | not detected | Yes |
| MR5VU_Rep2 | not detected | not detected | Yes |

**Supplementary Table 6** Fusion detection - intermediate precision (inter-operator, -lot, -run)

| **Sample ID** | **Library Batch** | **Operator** | **Reagent Lot** | **Expected Fusion** | **Observed Fusion** | **Concordant?** |
| --- | --- | --- | --- | --- | --- | --- |
| B1NVL | Batch 7 | 1 | Lot B | *TPM3::NTRK1* | *TPM3::NTRK1* | Yes |
| B1NVL | Batch 9 | 3 | Lot C | *TPM3::NTRK1* | *TPM3::NTRK1* | Yes |
| MR5VU | Batch 4 | 1 | Lot A | not detected | not detected | Yes |
| MR5VU | Batch 5 | 2 | Lot B | not detected | not detected | Yes |
| MR5VU Rep 1 | Batch 6 | 1 | Lot B | not detected | not detected | Yes |
| MR5VU Rep 2 | Batch 6 | 1 | Lot B | not detected | not detected | Yes |
| PB05R | Batch 7 | 1 | Lot C | *ETV6::NTRK3; NTRK3::ETV6* | *ETV6::NTRK3; NTRK3::ETV6* | Yes |
| PB05R | Batch 8 | 3 | Lot C | *NTRK3::ETV6* | *NTRK3::ETV6* | Yes |
| RNMWE | Batch 4 | 1 | Lot B | *EML4::NTRK3* | *EML4::NTRK3* | Yes |
| RNMWE | Batch 5 | 2 | Lot B | *EML4::NTRK3* | *EML4::NTRK3* | Yes |
| RNMWE Rep 1 | Batch 6 | 1 | Lot B | *EML4::NTRK3* | *EML4::NTRK3* | Yes |
| RNMWE Rep 2 | Batch 6 | 1 | Lot B | *EML4::NTRK3* | *EML4::NTRK3* | Yes |
| UWD23 | Batch 4 | 1 | Lot B | *EML4::ALK* | *EML4::ALK* | Yes |
| UWD23 | Batch 10 | 2 | Lot C | *EML4::ALK* | *EML4::ALK* | Yes |
| UWD23 | Batch 9 | 3 | Lot C | *EML4::ALK* | *EML4::ALK* | Yes |
| UWD23 | Batch 5 | 2 | Lot B | *EML4::ALK* | *EML4::ALK* | Yes |
| UWD23 Rep 1 | Batch 6 | 1 | Lot B | *EML4::ALK* | *EML4::ALK* | Yes |
| UWD23 Rep 2 | Batch 6 | 1 | Lot B | *EML4::ALK* | *EML4::ALK* | Yes |

**Supplementary Table 7** Fusion detection - intermediate precision (machine)

| **Sample ID** | **Fusions detected on Sequencer-1** | **Fusions detected on Sequencer-2** | **Concordant between sequencers?** |
| --- | --- | --- | --- |
| RNMWE | *EML4::NTRK3* | *EML4::NTRK3* | Yes |
| UWD23 | *EML4::ALK* | *EML4::ALK* | Yes |
| 6MV24 | *EML4::ALK* | *EML4::ALK* | Yes |
| 1KS6A | *NPM1::ALK* | *NPM1::ALK* | Yes |
| RYUGZ | not detected | not detected | Yes |
| PLBGS | not detected | not detected | Yes |
| 174DS | not detected | not detected | Yes |
| TX79T | not detected | not detected | Yes |
| E0DLZ | not detected | not detected | Yes |
| YUKWB | not detected | not detected | Yes |
| Fusion-positive reference RNA | *TPM3::NTRK1*  *EML4::ALK*  *QKI::NTRK2*  *ETV6::NTRK3* | *TPM3::NTRK1*  *EML4::ALK*  *QKI::NTRK2*  *ETV6::NTRK3* | Yes |
| Fusion-negative reference RNA | not detected | not detected | Yes |

**Supplementary Table 8** Fusion detection at varying RNA input amounts

| **Sample ID** | **RNA input** | **Expected Result** | **Observed Result** |
| --- | --- | --- | --- |
| B1NVL | 20 ng | *TPM3::NTRK1* | *TPM3::NTRK1* |
|  | 35 ng | *TPM3::NTRK1* | *TPM3::NTRK1* |
|  | 50 ng | *TPM3::NTRK1* | *TPM3::NTRK1* |
|  | 65 ng | *TPM3::NTRK1* | *TPM3::NTRK1* |
|  | 80 ng | *TPM3::NTRK1* | *TPM3::NTRK1* |
| PB05R | 20 ng | *ETV6::NTRK3* | *ETV6::NTRK3* |
|  | 35 ng | *ETV6::NTRK3* | *ETV6::NTRK3* |
|  | 50 ng | *ETV6::NTRK3* | *ETV6::NTRK3* |
|  | 65 ng | *ETV6::NTRK3* | *ETV6::NTRK3* |
|  | 80 ng | *ETV6::NTRK3* | *ETV6::NTRK3* |
| UWD23 | 20 ng | *EML4::ALK* | *EML4::ALK* |
|  | 35 ng | *EML4::ALK* | *EML4::ALK* |
|  | 50 ng | *EML4::ALK* | *EML4::ALK* |
|  | 65 ng | *EML4::ALK* | *EML4::ALK* |
|  | 80 ng | *EML4::ALK* | *EML4::ALK* |

**Supplementary Table 9** Fusion gene detection in fusion-negative/fusion-positive RNA mixes

| **Fusion-positive RNA ID** | **%fusion- positive RNA in mix** | **Expected Result** | **Observed Result** | **Supporting Reads** | **Concordance** |
| --- | --- | --- | --- | --- | --- |
| Positive-reference RNA | 100% | *TPM3::NTRK1*  *EML4::ALK*  *QKI::NTRK2*  *ETV6::NTRK3* | *TPM3::NTRK1*  *EML4::ALK*  *QKI::NTRK2*  *ETV6::NTRK3* | 1430  1242  995  169 | 4/4 |
|  | 25% | *TPM3::NTRK1*  *EML4::ALK*  *QKI::NTRK2*  *ETV6::NTRK3* | *TPM3::NTRK1*  *EML4::ALK*  *QKI::NTRK2*  *ETV6::NTRK3* | 404  375  274  69 | 4/4 |
|  | 10% | *TPM3::NTRK1*  *EML4::ALK*  *QKI::NTRK2*  *ETV6::NTRK3* | *TPM3::NTRK1*  *EML4::ALK*  *QKI::NTRK2*  *ETV6::NTRK3* | 197  154  142  27 | 4/4 |
|  | 5% | *TPM3::NTRK1*  *EML4::ALK*  *QKI::NTRK2*  *ETV6::NTRK3* | *TPM3::NTRK1*  *EML4::ALK*  *QKI::NTRK2*  *ETV6::NTRK3* | 94  81  75  21 | 4/4 |
|  | 1% | *TPM3::NTRK1*  *EML4::ALK*  *QKI::NTRK2*  *ETV6::NTRK3* | *TPM3::NTRK1*  *EML4::ALK*  *QKI::NTRK2* | 23  16  12 | 3/4 |
| PB05R | 100% | *ETV6::NTRK3* | *ETV6::NTRK3* | 293  14 | 1/1 |
|  | 25% | *ETV6::NTRK3* | *ETV6::NTRK3* | 65 | 1/1 |
|  | 10% | *ETV6::NTRK3* | *ETV6::NTRK3* | 29 | 1/1 |
|  | 5% | *ETV6::NTRK3* | *ETV6::NTRK3* | 11 | 1/1 |
|  | 1% | *ETV6::NTRK3* | not detected | N/A | 0/1 |
| UWD23 | 100% | *EML4::ALK* | *EML4::ALK* | 71 | 1/1 |
|  | 25% | *EML4::ALK* | *EML4::ALK* | 21 | 1/1 |
|  | 10% | *EML4::ALK* | *EML4::ALK* | 9 | 1/1 |
|  | 5% | *EML4::ALK* | not detected | N/A | 0/1 |
|  | 1% | *EML4::ALK* | not detected | N/A | 0/1 |

**Supplementary Table 10** Extrapolated fusion gene copy number in fusion-positive/fusion-negative reference RNA mixes

| **Fusion gene** | **RNA mix with minimum copy number detected** | ***Copy number in 50 ng** | ***Copy number in RNA mix** |
| --- | --- | --- | --- |
| *TPM3::NTRK1* | 1% fusion-positive reference RNA | 14,540 | 145.4 |
| *QKI::NTRK2* | 1% fusion-positive reference RNA | 31,254 | 312.54 |
| *EML4::ALK* | 1% fusion-positive reference RNA | 5,775 | 57.75 |
| *ETV6::NTRK3* | 5% fusion-positive reference RNA | 2,242 | 112.1 |

*Copy number input extrapolated from manufacturer’s ddPCR measurements.

**Supplementary Table 11** RNA sample properties for study of gDNA as an interferent

| **Sample ID** | **Baseline %gDNA** | **Expected fusion** |
| --- | --- | --- |
| RNMWE | 13.7% | *EML4::NTRK3* |
| UWD23 | 4.5% | *EML4::ALK* |
| 226BJ | 6.7% | none detected |

**Supplementary Table 12** Fusions detected for study of gDNA as an interferent

| **Sample ID** | **Expected Fusion Result** | **Observed Fusion Result** | **Concordant?** |
| --- | --- | --- | --- |
| RNMWE_0%_gDNA_spike-in | *EML4::NTRK3* | *EML4::NTRK3* | Yes |
| RNMWE_5%_gDNA_spike-in | *EML4::NTRK3* | *EML4::NTRK3* | Yes |
| RNMWE_10%_gDNA_spike-in | *EML4::NTRK3* | *EML4::NTRK3* | Yes |
| RNMWE_20%_gDNA_spike-in | *EML4::NTRK3* | *EML4::NTRK3* | Yes |
| RNMWE_30%_gDNA_spike-in | *EML4::NTRK3* | *EML4::NTRK3* | Yes |
| UWD23_0%_gDNA_spike-in | *EML4::ALK* | *EML4::ALK* | Yes |
| UWD23_5%_gDNA_spike-in | *EML4::ALK* | *EML4::ALK* | Yes |
| UWD23_10%_gDNA_spike-in | *EML4::ALK* | *EML4::ALK* | Yes |
| UWD23_20%_gDNA_spike-in | *EML4::ALK* | *EML4::ALK* | Yes |
| UWD23_30%_gDNA_spike-in | *EML4::ALK* | *EML4::ALK* | Yes |
| 226BJ_0%_gDNA_spike-in | not detected | not detected | Yes |
| 226BJ_5%_gDNA_spike-in | not detected | not detected | Yes |
| 226BJ_10%_gDNA_spike-in | not detected | not detected | Yes |
| 226BJ_20%_gDNA_spike-in | not detected | not detected | Yes |
| 226BJ_30%_gDNA_spike-in | not detected | not detected | Yes |

**Supplementary Table 13** Validation of OncoPrism-HNSCC fusion detection by orthogonal methods

| **SAMPLE ID** | **Cancer type** | **Expected fusion** | **Orthogonal testing method** | **Concordant? (Y/N)** | **Comments** |
| --- | --- | --- | --- | --- | --- |
| UWD23 | Lung | *EML4::ALK* | TSO500, ALK IHC, GatewaySeq | Y | GatewaySeq detected no fusions, but sample was positive for *EML4::ALK* fusion by TSO500 and positive for ALK fusion by IHC, and is therefore designated as concordant |
| 6MV24 | NSCLC | *EML4::ALK* | GatewaySeq, ALK IHC | Y |  |
| 73Z6P | NSCLC | *EML4::ALK* | GatewaySeq, ALK IHC | Y |  |
| COUCS | NSCLC | *EML4::ALK* | TSO500, GatewaySeq, ALK IHC | Y | GatewaySeq detected no fusions and sample failed ALK IHC QC, but was positive for *EML4::ALK* fusion by TSO500, and is therefore designated as concordant |
| O2USE | NSCLC | *EML4::ALK* | TSO500, GatewaySeq, ALK IHC | Y | GatewaySeq detected no fusions, but sample was positive for *EML4::ALK* fusion by TSO500 and positive for ALK fusion by IHC, so is therefore designated as concordant |
| ST6HK | NSCLC | *EML4::ALK* | GatewaySeq, ALK IHC | Y |  |
| U6DI7 | NSCLC | *EML4::ALK* | TSO500, GatewaySeq, ALK IHC | Y | GatewaySeq detected no fusions and sample failed ALK IHC QC, but was positive for *EML4::ALK* fusion by TSO500, and is therefore designated as concordant |
| WM11J | NSCLC | *EML4::ALK* | GatewaySeq, ALK IHC | Y |  |
| B1NVL | Colorectal | *TMP3::NTRK1* | TSO500, Pan-Trk IHC | Y |  |
| RNMWE | Colon | *EML4::NTRK3* | TSO500, GatewaySeq, Pan-Trk IHC | Y | Pan-Trk IHC was equivocal, but *EML4::NTRK3* fusion was confirmed by TSO500 and GatewaySeq |
| 1KS6A | NHL | *NPM1::ALK* | ALK IHC, GatewaySeq | Y |  |
| JRL2T | NHL | *CLTC::ALK* | ALK IHC, GatewaySeq | Y |  |
| PB05R | Head and Neck (SGC) | *ETV6:NTRK3* | ALK IHC, Pan-Trk IHC, GatewaySeq | Y |  |
| KXFRY | Head and Neck (Thyroid) | *ETV6:NTRK3* | GatewaySeq | Y |  |
| Y462C | Head and Neck (HNSCC) | not detected | ALK IHC, Pan-Trk IHC, GatewaySeq, STAR-Fusion | Y | Pan-Trk IHC was positive; NGS-based GatewaySeq was negative for *ALK, NTRK1, ETV6::NTRK3* fusions; sample RNA was negative by STAR-Fusion; probable Pan-Trk IHC false-positive |
| LQQUH | Head and Neck (HNSCC) | not detected | ALK IHC, Pan-Trk IHC, Star-Fusion | Y | Pan-Trk IHC was positive, but sample was QNS for additional orthogonal DNA testing; sample RNA was negative by STAR-Fusion; probable Pan-Trk IHC false-positive |
| 07H1V | Head and Neck (HNSCC) | not detected | ALK IHC, Pan-Trk IHC, GatewaySeq | Y |  |
| 1AMJ7 | Head and Neck (HNSCC) | not detected | ALK IHC, Pan-Trk IHC, GatewaySeq | Y |  |
| 226BJ | Head and Neck (HNSCC) | not detected | ALK IHC, Pan-Trk IHC, GatewaySeq | Y |  |
| 3HD42 | Head and Neck (HNSCC) | not detected | ALK IHC, Pan-Trk IHC, GatewaySeq | Y |  |
| 5AX1V | Head and Neck (HNSCC) | not detected | ALK IHC, Pan-Trk IHC, GatewaySeq | Y |  |
| 5ZW04 | Head and Neck (HNSCC) | not detected | ALK IHC, Pan-Trk IHC, GatewaySeq | Y |  |
| B0U4W | Head and Neck (HNSCC) | not detected | ALK IHC, Pan-Trk IHC, GatewaySeq | Y |  |
| I96PP | Head and Neck (HNSCC) | not detected | ALK IHC, Pan-Trk IHC, GatewaySeq | Y |  |
| J16T3 | Head and Neck (HNSCC) | not detected | ALK IHC, Pan-Trk IHC, GatewaySeq | Y |  |
| KD4VA | Head and Neck (HNSCC) | not detected | ALK IHC, Pan-Trk IHC, GatewaySeq | Y |  |
| KTL7F | Head and Neck (HNSCC) | not detected | ALK IHC, Pan-Trk IHC, GatewaySeq | Y |  |
| MR5VU | Head and Neck (HNSCC) | not detected | ALK IHC, Pan-Trk IHC, GatewaySeq | Y |  |
| RYUGZ | Head and Neck (HNSCC) | not detected | ALK IHC, Pan-Trk IHC, GatewaySeq | Y |  |
| ULR4R | Head and Neck (HNSCC) | not detected | ALK IHC, Pan-Trk IHC, GatewaySeq | Y |  |
| V7MWZ | Head and Neck (HNSCC) | not detected | ALK IHC, Pan-Trk IHC, GatewaySeq | Y |  |
| VS8KQ | Head and Neck (HNSCC) | not detected | ALK IHC, Pan-Trk IHC, GatewaySeq | Y |  |
| W69KR | Head and Neck (HNSCC) | not detected | ALK IHC, Pan-Trk IHC, GatewaySeq | Y |  |
| WDCLX | Head and Neck (HNSCC) | not detected | ALK IHC, Pan-Trk IHC, GatewaySeq | Y |  |
| 0OUOY | Head and Neck (HNSCC) | not detected | ALK IHC, Pan-Trk IHC | Y |  |
| 174DS | Head and Neck (HNSCC) | not detected | ALK IHC, Pan-Trk IHC | Y |  |
| 3Y1GY | Head and Neck (HNSCC) | not detected | ALK IHC, Pan-Trk IHC | Y |  |
| 4K9GH | Head and Neck (HNSCC) | not detected | ALK IHC, Pan-Trk IHC | Y |  |
| 51E2C | Head and Neck (HNSCC) | not detected | ALK IHC, Pan-Trk IHC | Y |  |
| 5GHP9 | Head and Neck (HNSCC) | not detected | ALK IHC, Pan-Trk IHC | Y |  |
| 6EDBL | Head and Neck (HNSCC) | not detected | ALK IHC, Pan-Trk IHC | Y |  |
| 79Y53 | Head and Neck (HNSCC) | not detected | ALK IHC, Pan-Trk IHC | Y |  |
| 7Z3F1 | Head and Neck (HNSCC) | not detected | ALK IHC, Pan-Trk IHC | Y |  |
| A15X0 | Head and Neck (HNSCC) | not detected | ALK IHC, Pan-Trk IHC | Y |  |
| DCL56 | Head and Neck (HNSCC) | not detected | ALK IHC, Pan-Trk IHC | Y |  |
| DUJQC | Head and Neck (HNSCC) | not detected | ALK IHC, Pan-Trk IHC | Y |  |
| E0DLZ | Head and Neck (HNSCC) | not detected | ALK IHC, Pan-Trk IHC | Y |  |
| H2AW8 | Head and Neck (HNSCC) | not detected | ALK IHC, Pan-Trk IHC | Y |  |
| HF89S | Head and Neck (HNSCC) | not detected | ALK IHC, Pan-Trk IHC | Y |  |
| IDUDB | Head and Neck (HNSCC) | not detected | ALK IHC, Pan-Trk IHC | Y |  |
| IPX5J | Head and Neck (HNSCC) | not detected | ALK IHC, Pan-Trk IHC | Y |  |
| J0BUV | Head and Neck (HNSCC) | not detected | ALK IHC, Pan-Trk IHC | Y |  |
| L851R | Head and Neck (HNSCC) | not detected | ALK IHC, Pan-Trk IHC | Y |  |
| ODL4U | Head and Neck (HNSCC) | not detected | ALK IHC, Pan-Trk IHC | Y |  |
| OPB1Q | Head and Neck (HNSCC) | not detected | ALK IHC, Pan-Trk IHC | Y |  |
| PLBGS | Head and Neck (HNSCC) | not detected | ALK IHC, Pan-Trk IHC | Y |  |
| PLZTE | Head and Neck (HNSCC) | not detected | ALK IHC, Pan-Trk IHC | Y |  |
| PZY1K | Head and Neck (HNSCC) | not detected | ALK IHC, Pan-Trk IHC | Y |  |
| QFPJW | Head and Neck (HNSCC) | not detected | ALK IHC, Pan-Trk IHC | Y |  |
| QP4DC | Head and Neck (HNSCC) | not detected | ALK IHC, Pan-Trk IHC | Y |  |
| QR103 | Head and Neck (HNSCC) | not detected | ALK IHC, Pan-Trk IHC | Y |  |
| R26TL | Head and Neck (HNSCC) | not detected | ALK IHC, Pan-Trk IHC | Y |  |
| SUU9G | Head and Neck (HNSCC) | not detected | ALK IHC, Pan-Trk IHC | Y |  |
| TX79T | Head and Neck (HNSCC) | not detected | ALK IHC, Pan-Trk IHC | Y |  |
| UXTBE | Head and Neck (HNSCC) | not detected | ALK IHC, Pan-Trk IHC | Y |  |
| WN52W | Head and Neck (HNSCC) | not detected | ALK IHC, Pan-Trk IHC | Y |  |
| YUKWB | Head and Neck (HNSCC) | not detected | ALK IHC, Pan-Trk IHC | Y |  |

**Supplementary Table 14** OncoPrism-HNSCC fusion-detection compared to orthogonal methods

| **Accuracy (95% CI)** | **Negative percent agreement (95% CI)** | **Positive percent agreement (95% CI)** |
| --- | --- | --- |
| 100% (94.6–100%) | 100% (93.3–100%) | 100% (76.8–100%) |
